# RASTA-Bacteria: a web-based tool for identifying toxin-antitoxin loci in prokaryotes

**DOI:** 10.1186/gb-2007-8-8-r155

**Published:** 2007-08-01

**Authors:** Emeric W Sevin, Frédérique Barloy-Hubler

**Affiliations:** 1 CNRS UMR6061 Génétique et Développement, Université de Rennes 1, IFR 140, Av. du Prof. Léon Bernard, CS 34317, 35043 Rennes, France; 2 CNRS UMR6026 Interactions Cellulaires et Moléculaires, Groupe DUALS, Université de Rennes 1, IFR140, Campus de Beaulieu, Av. du Général Leclerc, 35042 Rennes, France

## Abstract

Toxin/antitoxin (TA) systems, viewed as essential regulators of growth arrest and programmed cell death, are widespread among prokaryotes, but remain sparsely annotated. We present RASTA-Bacteria, an automated method allowing quick and reliable identification of TA loci in sequenced prokaryotic genomes, whether they are annotated open reading frames or not. The tool successfully confirmed all reported TA systems, and spotted new putative loci upon screening of sequenced genomes. RASTA-Bacteria is publicly available at .

## Abstract 2

RASTA-Bacteria is an automated method that allows quick and reliable identification of toxin/antitoxin loci in sequenced prokaryotic genomes, whether they are annotated Open Reading Frames or not.

## Rationale

More than 500 prokaryotic genomes have now been completely sequenced and annotated, and the number of sequencing projects underway (approximately 1,300) indicates that the amount of such data is going to rise very rapidly [1,2]. Large-scale comparative genomics based on these data constituted a giant leap forward in the process of gene identification. Nevertheless, substantial numbers of annotated open reading frames (ORFs) throughout the sequenced genomes remain hypothetical, most of which are 200 amino acids in length or shorter [3]. Luckily, interest in these small ORFs (sORFs) is growing [4], and recent work in *Sacharromyces cerevisiae *shows that they may be involved in key cellular functions [5].

The toxin/antitoxin (TA) modules are a group of sORFs for which knowledge has been improving over the past two decades. Most TA modules are constituted of two adjacent co-oriented but antagonist genes: one encodes a stable toxin harmful to an essential cell process, and the second a labile antitoxin that blocks the toxin's activity by DNA- or protein-binding [6]. TA pairs have been classified into two types. The first are those where the antitoxin is an antisense-RNA. They have been linked to plasmid stabilization by means of a post-segregational killing (PSK) effect, [7] (for a review, see [8]). The second type, on which we focus in this study, includes loci where the antitoxin is a fully translated protein. For consistency with previous studies, we shall refer to them throughout this paper as TA systems.

For some time after their discovery in 1983 [9], TA systems were only found on plasmids. They were defined as plasmid inheritance guarantor systems, and called 'plasmid addiction systems'. Several years later, two homologous TA operons were discovered on the *Escherichia coli *chromosome [10,11]. Interest in these chromosomal TA systems led to the discovery of further systems in various bacteria [12-14], and of their involvement in programmed cell death (PCD) [15]. It was suggested that under severe starvation conditions, the TA-mediated PCD of moribund subpopulations provides the remaining healthy cells with nutrients, thus benefiting the species. Proof was later established that some TA systems actually provoke a static state in certain adverse conditions, in which cells remain viable but do not proliferate, and that this state is fully reversible on cognate antitoxin induction [16]. However, it was later shown that this reversible effect is only possible within a limited time frame. Subsequently, there is a 'point of no return' in the killing effect of the toxin [17,18].

TA systems, widespread among both bacteria and archaea [19], are currently classified into eight families, depending on their structural features or modes of action [20]. Little is known about the only three-component family, whose founding member is the omega-epsilon-zeta (ω-ε-ζ) system from plasmid pSM19035, except that the additional gene (ω) acts as a repressor regulating the transcription of the operon [21]. ω-ε-ζ systems are found only in Gram-positive bacteria. The remaining seven, two-component families, include: the ParDE system, found in Gram-negative and Gram-positive bacteria and in archaea, targets DNA gyrase [22]; HigBA, unique in that its toxin is located upstream from its antitoxin [23], is found in Gram-negative and Gram-positive bacteria, and its action involves mRNA cleavage [24]; the *phd*/*doc *locus, found in all types of prokaryotes, is believed to inhibit translation [25]; and the *vapBC *locus, found both on plasmids and chromosomes, seems to be the TA system with the highest copy-number in the prokaryotes that bear them, but no cellular target has yet been reported, although VapC toxins contain a PIN domain (homologue of the pilT amino-terminal domain: ribonuclease involved in nonsense-mediated mRNA decay and RNA interference in eukaryotes), suggesting that the system may contribute to quality control of gene expression [26]. The other three families are the best characterized: the *ccdAB *locus, found only in some Gram-negative bacteria, stabilizes plasmids upon replication by targeting DNA gyrase [27]; members of the RelBE family, present in Gram-negatives, Gram-positives and archaea, inhibit cell growth by impairing translation due to mRNA cleavage through the A-site of the ribosome [28,29]; and finally, the toxins of the MazEF/PemIK family, sometimes referred to as 'RNA interferases' [30], are ribonucleases that cleave cellular mRNA, thus depriving the ribosomes of substrates to translate [31] - they have been found in Gram-negative and Gram-positive bacteria.

The role of TA systems in programmed cell death opens promising possibilities for the design of a new class of antibiotics [32]. Moreover, chromosome-borne TA systems are activated by various extreme conditions, including the presence of antibiotics [33] or infecting phages [34], thymine starvation or other DNA damage [35], high temperatures, and oxidative stress [36]. Their involvement in the response to amino acid starvation [37] also raises large interest: indeed, TA modules are believed to provide a backup system to the stringent response by controlling superfluous macromolecular biosynthesis during stasis independently of ppGpp [38], the stringent response alarmone eliciting the protective reactions cascade. A reduced rate of translation is associated with fewer translational errors, so TA loci may contribute to quality control of gene expression, helping the cells cope with nutritional stress [20]. Therefore, it remains a priority to exhaustively identify TA loci in prokaryotic organisms in order to improve our understanding of these systems and more broadly of the cellular mechanisms behind bacterial adaptation.

In 2005, Pandey and co-workers [39] performed an exhaustive search in 126 completely sequenced genomes (archaea and eubacteria), using standard sequence alignment tools (BLASTP and TBLASTN). Their work highlighted a surprising diversity in the distribution of TA loci: some organisms have many (*Nitrosomonas europaea *has 45 potential TA systems), whereas more than half of the other species have between 1 and 5, and 31 have none. Nevertheless, the use of basic nucleic or amino acid sequence similarity limits these findings to toxins and antitoxins for which a clear homolog exists; there is, therefore, a possible bias in their results. In view of the aforementioned lack of annotation of the small ORFs, and to improve localization techniques for TA systems, we developed a simple method for identifying all potential TA systems in a given bacterial genome: Rapid Automated Scan for Toxins and Antitoxins in Bacteria (RASTA-Bacteria). This method is based on the genomic features associated with toxins and antitoxins and the existence of conserved functional domains. The results, sorted by a confidence score, discard no candidate, thus providing the user an extensive overview of the data.

## Process overview

**Figure 1:**
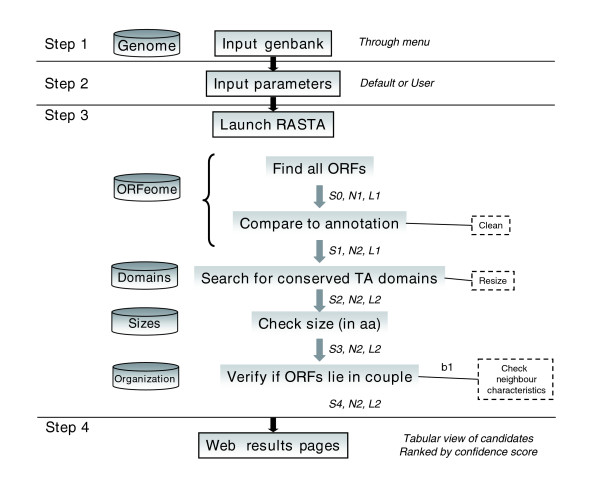
Schematic modular pipeline of RASTA-Bacteria. Step 1: provide a nucleic genome sequence in GenBank or raw Fasta format. Step 2: tune the search parameters (optional). Step 3: launch the search; each module calculates a local score, and possibly modifies the dataset (Sx = score at level x; Ny = number of ORFs in dataset; Lz = length distribution of dataset; b1 = bonus). Step 4: output in webpage and/or results files available for download.

The module-based pipeline of RASTA-Bacteria is described in Figure 1. The first step is to provide a genomic sequence. Even though it can be useful to test relatively short 'raw' nucleic sequences for the presence of a TA system, RASTA-Bacteria was designed to function with whole-replicon genomic sequences, regardless of their size (small plasmids or large chromosomes). The tool can thus take both simple (FASTA-formatted) nucleic sequences or fully annotated (GenBank) files as input data. They can either be selected from an extensive list of sequenced bacterial and archaeal genomes, or be provided by the user in the case of an unpublished genome.

**Figure 2:**
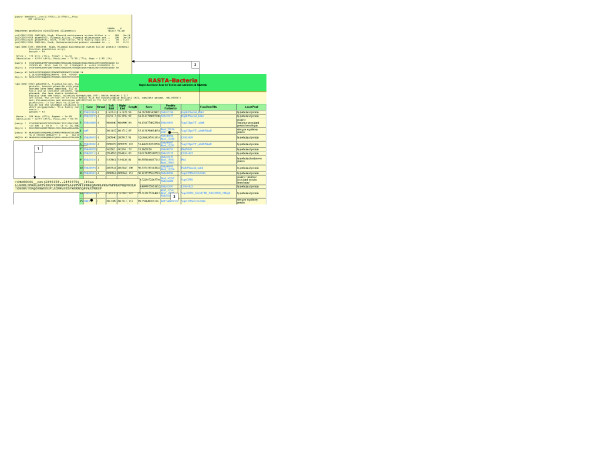
Screenshot of the results displayed as a webpage. This illustration shows the output results ranked by confidence score. The arrows represent internal links to additional supporting data. The amino acid sequence corresponding to an ORF as annotated by RASTA-Bacteria is shown (1). When a conserved TA domain was predicted, the alignment results can be seen in rpsblast output format (2). Anchor links between co-localized candidates allow checking for possible parity (3).

The second step enables the user to tune optional parameters for the search: depending on the origin of the input sequence, it is possible to choose the length-scoring model, from 'general', 'archaea', 'Gram+', and 'Gram-', on which the scoring function must rely. The sensitivity of the tool can also be improved by modifying the bit-score threshold for the RPSBLAST alignments. However, we defined the default value from our experiments and believe it is the most appropriate. Similarly, a minimal ORF size for the ORF finder can be defined, as well as an annotated gene overlap percentage threshold when verifying the annotation. These parameters limit the amount of data (hence time of computation), and should be refined only in particular cases, such as for known high-overlapping genomes for example. The third step is the run phase, performed as follows: first, screening of the nucleic sequence for open-reading frames; second, screening of newly determined ORFs for the presence of TA domains; third, size-based scoring of the ORFs; and fourth, scoring based on the pairing possibility of an ORF with another. In the last step, the results are combined to calculate a global confidence score for each ORF. These are then ranked accordingly and displayed to the user in a tabular format, which ensures clear visualization of the results and allows easy verification by cross-linkage to the data files. For raw nucleic sequences and files below 500 kb, the table is directly viewable in the user's web browser (Figure 2). The results table and supporting files are then available for download as a tar archive. For fully annotated genomes and files over 500 kB, no interactive display will be produced, and the user will be notified by email when the job ends that the archive is ready for download.

The method developed was automated using Perl, with sequence processing relying on the BioPerl library [40]. The script is embedded in a PHP-based web-interface. RASTA-Bacteria is publicly available from the application website [41].

## Description of the algorithm

### Genomic features used for discriminating TA systems

It should be noted here that *hipBA *loci (found to have a role in the production of 'persister cells' in *E. coli *[42]), as well as restriction-modification (type II) systems, can also be considered as TA systems. Nevertheless, the latter have been extensively identified and characterized elsewhere [43,44], and have been excluded from our work. Because of its specific organization, the three-component TA family (ω-ε-ζ) was also excluded from the present study.

**Figure 3:**
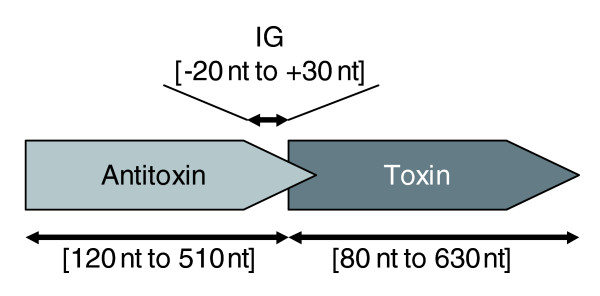
General genetic context of a TA loci. The typical TA loci organization with sizes and distance profiling is shown.

TA systems by definition consist of, at least, two genes: the 'dormant guard' role is fulfilled by the presence of a toxic and a protective protein together, although some orphan genes (for which conservation of functionality as such remains unclear) have been reported [39,45]. Whether or not the TA pairs are encoded by genes forming an operon, the spacer seldom extends beyond 30 nucleotides, and a small overlap (1 to 20 nucleotides in general) is the most common structure. The order of the two cooperating genes is also well conserved, with the antitoxin being upstream (Figure 3), although there is an exception: in *higBA *loci the toxin is upstream of the antitoxin [23].

**Figure 4:**
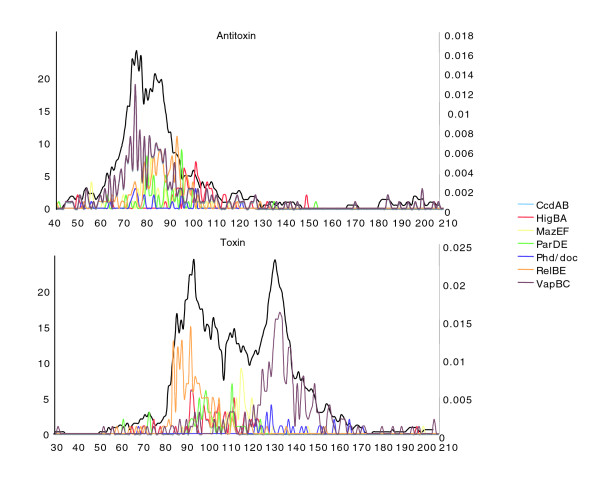
Length distribution of Bacterial toxins and antitoxins. The graph represents the length distribution of antitoxins and toxins in 126 organisms (from [39]), depending on their classification (X-axis, length in amino acids; left Y-axis, number of sequences). The black curves represent the probability over the total population (1,378 TA) for a sequence of length X to constitute a TA (right Y-axis), and were used to determine the length-criterion scoring function as described in the text.

TA genes in all prokaryotic species are small. According to Pandey *et al*. [39], antitoxins are 41 to 206 amino acids long and toxins 31 to 204 amino acids long, antitoxins generally being shorter than their partner toxins (Figure 4). Here too there seems to be an exception: the toxin of the HipBA system is 440 amino acids in length (not shown).

These two features have been used with success as preliminary filters to a biological search for unidentified TA pairs in *E. coli *[46], but this approach is too permissive to be accurate as an automatic predictor. By adding a third criterion, namely the presence of a conserved functional domain, the selectivity of the method over the input space can be improved. Furthermore, as the knowledge base of TA systems grows, sequence homology can provide further information.

### ORF detection and filtering

To bypass the mis-annotation of TA genes, which, like many small ORFs, are easily omitted during the annotation process, the tool begins with a naïve ORF prediction. This first step is essential to ensure that the analysis leaves no possible ORF aside. RASTA-Bacteria thus starts by predicting the entire set of valid ORFs in the sequence, defined as the series of triplets occurring between one of the four accepted prokaryotic start codons (NTG), and one of the three stop codons (TGA, TAG, TAA), with no further assumption about the profile of the ORF. In the case of alternative start codons, redundancy is avoided by considering only the longest possible sequence. Although no possible ORFs should be overlooked, existing genomic information (in the case of an annotated genome, the preferred input) should not be ignored. Indeed, even if sometimes flawed, the original annotation can provide RASTA-Bacteria with valuable hints. Therefore, the tool recovers all the annotated features of the sequence, and compares the 'naïve' ORFs to the existing set of genes. If a naïve ORF overlaps an annotated gene (whose 'product' and 'confidence' fields do not display the terms 'unknown', 'putative', or 'hypothetical') by more than a threshold percentage (see parameters), then it is discarded as a spurious ORF. If the considered ORF corresponds to an annotated ORF, its score is rewarded to reflect the annotators' work, that is, the probability that this ORF actually encodes a protein. For reasons of consistency, this process also renames existing ORFs with their common designation.

### Conserved domain verification: a specific TA-dedicated database

Once the whole list of candidate ORFs is established, the ORFs undergo a conserved domain search. To achieve this, we use the Reverse PSI-BLAST program (RPSBLAST, part of the standalone blast archive, release 2.2.14 [47]), which searches a query sequence against a database of pre-computed lookup tables called PSSMs (position specific scoring matrices), originating from the Pfam, Smart, COG, KOG and cd alignment collections (the complete archive of conserved domain PSSMs can be found at [48]). These profiles then need to be formatted as a usable database by the formatrpsdb tool [47]. For our purposes, we thus built a dedicated TA conserved domains database (TAcddb), compiled from the existing profiles of domains known to belong to toxin and antitoxin genes (Table 1), against which all the sequences in amino acids are searched. Consequently, TA systems with unknown functionally conserved domains are unfortunately liable to be penalized. However, the combining of different criteria tempers the risk of overlooking them, and the database is able to evolve as it can be re-compiled with any new set of PSSMs.

**Table 1:** List of PSSM profiles selected in *TAcddb *to verify the presence of a conserved TA-related domain

PSSMid	CD accession name	Relation/involvement in TA world	Reference
28977	cd00093-HTH_XRE	XRE-like domain present in HigA and VapB antitoxins	[20], this study
31586	COG1396-HipB	Involved in production of persister cells (antitoxin)	[20]
31676	COG1487-VapC	Quality control of gene expression	[57]
31786	COG1598	HicB of HicAB system (function undetermined)	[58]
31910	COG1724	HicA of HicAB system (function undetermined)	[58]
32033	COG1848	PIN domain, present in VapC toxins	[20,59,60]
32185	COG2002-AbrB	Domain present in of MazE and VapB antitoxins	[20]
32209	COG2026-RelE	Toxin of cytotoxic translational repressor system	[14,28,29]
32344	COG2161-StbD	Antitoxin of the RelBE family	[61]
32487	COG2336-MazE	Growth regulator (antitoxin)	[45]
32488	COG2337-MazF	Growth inhibitor (toxin)	[45]
32907	COG3093-VapI	Named from VapI region; corresponds to VapB antitoxins (Plasmid maintenance)	[62], this study
33351	COG3549-HigB	Toxin of plasmid maintenance system	[23]
33352	COG3550-HipA	Involved in production of persister cells (toxin)	[20]
33408	COG3609	CopG/Arc/MetJ DNA-binding domain, present in RelB, ParD, VapBCand CcdA antitoxins	[20], this study
33452	COG3654-Doc	Toxin of probable translational inhibitor system	[25,63]
33466	COG3668-ParE	Toxin of plasmid stabilization system	[22,64]
33870	COG4113	PIN domain, present in VapC toxins	[20,59,60]
33875	COG4118-Phd	Antitoxin to translational inhibitor Doc	[65]
33951	COG4226-HicB	HicB of HicAB system (predicted)	[58]
34119	COG4423	Predicted antitoxin of PIN domain toxins (VapC)	[57,60]
34135	COG4456-VagC	Antitoxin of plasmid maintenance system	[66]
34307	COG4691-StbC	Plasmid stability proteins (HigBA family)	[67,68], this study
34891	COG5302-CcdA	Antitoxin of plasmid stabilization system	[27,69]
35058	COG5499	Predicted transcription regulators with HTH domain	[20], this study
41431	pfam01381-Hth_3	Present in antitoxins of HigBA and VapBC families	[20], this study
41452	pfam01402-Hth_4	Present in CopG repressors (RelBE, ParDE, VapBC, and CcdAB families)	[20], this study
41869	pfam01845-CcdB	Toxin of plasmid stabilization system	[69]
41874	pfam01850-PIN	DNA binding PIN domain, present in VapC toxins	[59,60]
42429	pfam02452-PemK	Toxin of the MazEF family	[70]
43931	pfam04014-AbrB	Domain present in MazE and VapB antitoxins	[20], this study
44135	pfam04221-RelB	Antitoxin to translational repressor RelE	[14]
44915	pfam05012-Doc	Toxin of probable translational inhibitor system	[63]
44918	pfam05015-Plasmid_killer	Toxins of the HigBA family	[23], this study
44919	pfam05016-Plasmid_stabil	Toxins of the RelE family	[14], this study
45431	pfam05534-HicB	Member of the HicAB system	[58]
47246	pfam07362-CcdA	Antitoxin of plasmid stabilization system	[27,69]
47831	smart00530-Xre	XRE-like HTH domain present in HigA and VapB	[20], this study

References to 'this study' correspond to domains found in this study upon sequence analysis of described TA candidates. AbrB, AidB regulator; HTH, helix-turn-helix; PIN, homologues of the pilin biogenesis protein pilT amino-terminal domain; XRE, xenobiotic response element.

For each candidate, the hits are analyzed to select the most likely in terms of both homology and sequence alignment length. If the candidate ORF exhibits a clear homology, namely a high score and over 80% of a full product domain aligned, but is longer than the corresponding profile, it is scanned for alternative start codons to identify any other 5' end that gives a better profile fit. If this is the case, the ORF is resized to its new coordinates. A short description of the possible domain is stored for subsequent display as a hint to the user for further classification, with an internal hyperlink to the alignment: again, no information is discarded and all the results can be visually assessed. Here, each reference domain used is levelheaded with a coefficient representing its implication in the TA kingdom: those defined by a confirmed TA family have a higher coefficient than domains found in TAs but not exclusive to them (for example, PIN versus VapB domain). This coefficient is computed together with the alignment data to yield the 'domain score'.

### The length criterion

The candidates proceed to a size-scoring module. Based on the lengths of 1,378 TA sequences (Figure 4) described following the extensive search by Pandey's team [39], we calculated the probability for length l of a candidate to be that of a toxin or an antitoxin as follows:

$$P(L=l)=\frac{n_{l}}{N}$$

where *N *= 1,378. We then defined our scoring function by averaging the probability over k neighboring lengths before and after the considered length such that:

$$f(l)=\frac{1}{2k+1}\sum_{i=-k}^{k}P(L=l+i)$$

This smoothes the curb of probabilities to some extent, as it avoids accidental high or low counts of a given length to be given undue weight with respect to surrounding lengths. Several datasets were created so that the scoring function reflects the different types of organisms: general, archaea, Gram-negative and Gram-positive. The user can thus choose which model to use depending on the species being considered. Similarly, although defining size functions for each of the seven TA families is at first sight appealing, it should be emphasized that automatic classification of TA loci is risky. This is due to diverging homologies: some toxin motifs pair with antitoxin motifs, or more simply toxins/antitoxins of a given family sometimes demonstrate similarity with those of another family [39]. Therefore, relying on such specific characteristics for the size criterion evaluation might lead to mis-scoring.

### ORF 'pair organization' scoring criterion

Finally, the method verifies that the ORFs are paired on the strand considered. To do so, the module searches for close neighbors upstream and downstream of the ORF, in agreement with the distance parameter described above: a neighbor is considered close if it lies less than 30 base-pairs away from the extremities of the ORF, and if it overlaps the ORF by less than 20 base-pairs. In practice, both values can be somewhat enlarged, so as to avoid potential loss of candidates in the case of an extended span of the ORF due to alternative start codons. Thus, if an ORF fits these criteria, its score is rewarded. Furthermore, if the neighbor exhibits a TA length and/or a TA domain, the score is given the corresponding bonus. Obviously, this diminishes the chances of fortuitous or clearly non-TA characterized operons finding themselves among the top candidates.

## RASTA-Bacteria in action

All tests reported in this section were carried out with annotated .gbk files downloaded on 1 September 2006 from the RefSeq repository [49], on a Mac PowerPC G5 with Mac OS X v.10.3.9. For multi-replicon organisms, all episomes were included in the analysis. Running times were between 40 s (for a 600 Mb genome) and 33 minutes (for a 9 Gb genome).

### Application to the alpha-proteobacteria model: *Sinorhizobium meliloti*

*S. meliloti *is a Gram-negative alpha-proteobacterium studied in our laboratory that is found both free-living in soil and in a symbiotic interaction with alfalfa where it forms root nodules. Its genome is made up of a 3.65 Mb circular chromosome and two essential megaplasmids, pSymA (1.35 Mb) and pSymB (1.68 Mb), all of them being GC rich (62.2% global) [50]. These features (large and tripartite genome with recently acquired plasmid, free and symbiotic life ability) make *S. meliloti *an interesting model for the validation of RASTA-bacteria. In the 2005 search by Pandey *et al*. [39], 12 TA systems (2 *relBE*-like, 3 *higBA*-like, and 7 *vapBC*-like) were identified, but only the chromosome was considered. We analyzed all three replicons with RASTA-Bacteria, as they are all constituents of the complete genome. Of the 12 systems identified by Pandey *et al*., 11 were positively discriminated by RASTA, including the ntrPR operon, which was recently shown to function as a TA system [51], demonstrating the good accuracy of our software. The 12th one (higBA-2, GI15965582-15965583) was only poorly rewarded by the method described here; indeed, none of the TA domain profiles corresponding to its described classification (nor others) were matched by the members of this TA pair, which furthermore do not fit the size and distance criteria. Further sequence analysis did reveal similarity with a putative addiction module killer protein for the amino-terminal half of gene 15965582, but a second conserved domain in its carboxy-terminal half, as well as the conserved domains ('ABC transporter') found in its reported partner, are rather contradictory with the fact that this pair might comprise a valid TA system. There is thus no concrete evidence that enables us to confirm this hypothesis.

**Figure 5:**
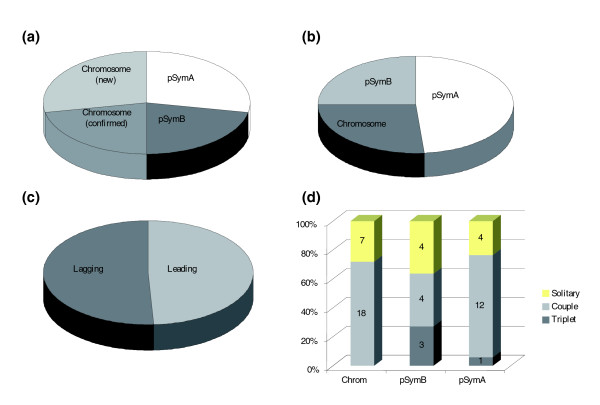
TA loci features in individual replicons of *S. meliloti *strain 1021. **(a) **Repartition of TA loci in the chromosome (new and confirming Pandey *et al*.'s [39] findings) and in the two megaplasmids. **(b) **Percentage of TA loci as a function of replicon size. **(c) **Repartition with respect to leading and lagging strands of replication. **(d) **Frequency of the three genomic organizations found for TA genes in the three replicons.

**Figure 6:**
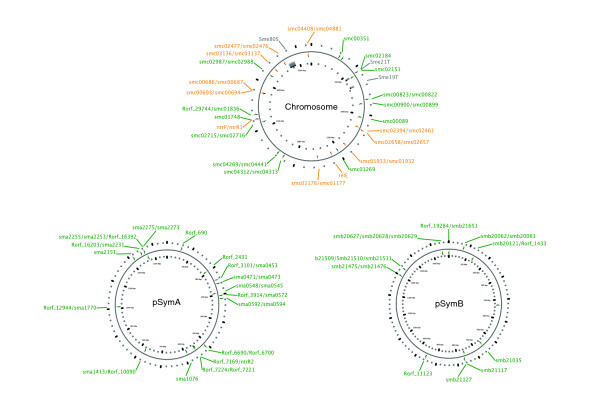
Maps of TA loci in individual replicons of *S. meliloti *strain 1021. The maps were created using CGView [53,54]. Green labels represent newly annotated TA genes, and orange labels represent RASTA-Bacteria predicted TA genes previously reported by Pandey *et al*. [39] On the chromosome, the grey SmeXXX regions correspond to genomic islands as described in the Islander database [55,56].

We found 14 additional putative TA loci on the chromosome (bringing the population to 25 for this replicon), 17 loci on pSymA and 11 on pSymB (Figure 5a). Hence, our approach predicts a total of 53 TA loci in the complete genome of *S. meliloti*, including 95 genes of which 18 are newly identified. Their distribution across the various replicons seems random, although there is an apparent alternation of rich and poor areas, in particular in the megaplasmids (Figure 6). Similarly, they are remarkably evenly distributed between lagging and leading strands (Figure 5c). Relative to the sizes of the replicons, megaplasmid A, suspected to have been acquired more recently in the genome, contains twice as many TA loci as the other replicons (Figure 5b). Interestingly, the genetic organizations are diverse, although pairs remain the most frequent (71.5 %): 12 genes in 4 triplets, 68 genes in 34 pairs and 15 solitary genes (12 encode antitoxins and 3 encode toxins, one of them being the chromosomal *relE*; Figure 4d).

The classification of candidates into families according to sequence homology alone is a tedious task. Nevertheless, it seems the two major families are *vapBC*, consistent with the findings of Pandey *et al*. [39], and parDE. No *ccdAB *locus was found, but the results indicate there may be *parDE *and *phd/doc *members (distributed on all three replicons) among the candidates, as well as one *mazEF *pair, situated on plasmid B.

### RASTA-bacteria results compared to those from previous studies

Our tool proved to be efficient and fast for the bacterium *S. meliloti*, which was used for its design. The effectiveness of RASTA-Bacteria for other sequences was first assessed using 14 prokaryotes previously studied by Pandey *et al*. [39] (Table 2): three gamma-proteobacteria (*E. coli *as an AT-rich generic model, *Coxiella burnetii *as an obligate host-associated organism and *Pseudomonas aeruginosa *as a free living, GC-rich bacterium); two alpha-proteobacteria (*Bradyrhizobium japonicum*, which has a large chromosome with significant horizontal rearrangements, and *Agrobacterium tumefaciens*, which has both circular and linear chromosomes); the genome with the largest predicted set of TA loci (*Nitrosomonas europeae *[39]); free-living firmicutes (*Lactococcus lactis*, *Bacillus*); one epsilon-proteobacteria (*Campylobacter jejuni*); three obligate host-associated organisms (*Rickettsia prowazekii*, *Buchnera aphidicola*, and *Mycobacterium leprae *for which Pandey *et al*. did not find any TA loci); and members of the Aquificae and Thermatogae extreme-life phylum (*Thermotoga maritima*, *Aquifex aeolicus*). Also, to assess the range of applicability of our tool, we tested the archaeum *Sulfolobus tokodaii*. The result files for all these species as well as for *S. meliloti *are available in the 'Pre-computed Data' section of our website [41].

**Table 2:** Results for 14 previously studied organisms

Organism	*ccdAB*	*higBA*	*mazEF*	*parDE*	*phd/doc*	*relBE*	*vapBC*	*hipBA*	Unclass.	Total
Aquifex aeolicus VF5*	0 (0)	0 (0)	0 (0)	0 (0)	0 (0)	1 (0)	6 (2)	2 (0)	0	9 (2)
A. tumefaciens str. C58*	0 (0)	0 (0)	1 (1)	3 (3)	1 (0)	7 (7)	5 (3)	6 (0)	1	24 (14)
*Bacillus anthracis Ames*	0 (0)	0 (0)	1 (1)	0 (0)	0 (0)	0 (0)	0 (0)	0 (0)	0	1 (1)
*Bacillus subtilis*	0 (0)	0 (0)	1 (1)	0 (0)	0 (0)	0 (0)	0 (0)	0 (0)	0	1 (1)
*Bradyrhizobium japonicum*	0 (0)	4 (4)	0 (0)	0 (0)	0 (0)	0 (0)	1 (1)	6 (0)	0	12 (5)
*Borrelia afzelii Pko*	0 (0)	0 (0)	0 (0)	0 (0)	0 (0)	0 (0)	0 (0)	0 (0)	0	0 (0)
*Buchnera aphidicola str.*	0 (0)	0 (0)	0 (0)	0 (0)	0 (0)	0 (0)	0 (0)	0 (0)	0	0 (0)
*Campylobacter jejuni*	0 (0)	0 (0)	0 (0)	0 (0)	0 (0)	1 (0)	1 (0)	0 (0)	0	2 (0)
*C. pneumoniae CWL029*	0 (0)	0 (0)	0 (0)	0 (0)	0 (0)	0 (0)	0 (0)	0 (0)	0	0 (0)
Coxiella burnetii RSA 493*	0 (0)	3 (3)	0 (0)	1 (1)	0 (0)	1 (1)	2 (2)	0 (0)	3	10 (7)
*Escherichia coli K12*	0 (0)	1 (1)	2 (2)	0 (0)	0 (0)	4 (3)	2 (0)	0 (0)	0	10 (6)
*Haemophilus ducreyi*	0 (0)	0 (0)	0 (0)	0 (0)	0 (0)	0 (0)	0 (0)	0 (0)	0	0 (0)
*Lactococcus lactis*	0 (0)	0 (0)	0 (0)	0 (0)	0 (0)	0 (0)	0 (0)	1 (0)	6	7 (0)
*Mycobacterium leprae TN*	0 (0)	0 (0)	0 (0)	0 (0)	0 (0)	0 (0)	0 (0)	0 (0)	0	0 (0)
*Mycoplasma genitalium*	0 (0)	0 (0)	0 (0)	0 (0)	0 (0)	0 (0)	0 (0)	0 (0)	0	0 (0)
*Nitrosomonas europaea*	1 (1)	8 (7)	5 (5)	6 (6)	2 (2)	10 (10)	20 (14)	1 (0)	4	57 (45)
*Prochlorococcus marinus*	0 (0)	0 (0)	0 (0)	0 (0)	0 (0)	0 (0)	0 (0)	0 (0)	0	0 (0)
*Pseudomonas aeruginosa*	0 (0)	5 (1)	0 (0)	1 (1)	1 (0)	1 (1)	0 (0)	2 (0)	3	13 (3)
*Rickettsia prowazekii*	0 (0)	0 (0)	0 (0)	0 (0)	0 (0)	0 (0)	0 (0)	0 (0)	0	0 (0)
Sinorhizobium meliloti*	0 (0)	6 (3)	1 (0)	8 (0)	3 (0)	2 (2)	27 (7)	1 (0)	5	53 (12)
*Sulfolobus tokodaii*	0 (0)	0 (0)	0 (0)	0 (0)	3 (3)	4 (4)	29 (25)	1 (0)	0	37 (32)
*Thermotoga maritima*	0 (0)	0 (1)	0 (0)	0 (0)	0 (0)	1 (0)	0 (0)	0 (0)	0	2 (1)

Numbers stand for TA systems (singleton or doublet) as predicted by: RASTA-Bacteria (numbers in parentheses are as predicted by Pandey *et al*. [39]). *Plasmids were not included in the analysis by Pandey *et al*. [39]. Unclass., unclassified.

RASTA-bacteria identified all TA loci previously predicted by Pandey *et al*. except for one locus in *S. meliloti *(see above) and one *higBA *system in *B. japonicum*, which was not retained because the confidence score was too low (although there are conserved domains, they are ambiguous and were not included in TAcddb). The absence of detectable TA genes from the three obligate host-associated organisms tested (*R. prowazekii*, *B. aphidicola*, *M. leprae*) was confirmed, as was the presence of a single TA locus in *Bacillus *sp. Our tool was more sensitive than the previously used method: in all other tested genomes, RASTA-Bacteria identified a large number of new candidate loci. This was largely due to detection of potential members of the *higBA*, *relBE*, *hipBA *families and especially the *vapBC *family. For example, even in the case of the well-documented model *E. coli*, RASTA-Bacteria predicts at least four new TA pairs with high confidence (*yfeD/yfeC*, *yafN/yafO*, *ygjN/ygjM *and *sohA/yahV*). In addition, the *ygiT/b3022*, *ydcQ/yncN *and *ydaS/ydaT *loci have at least one member with a conserved domain commonly found in antitoxins, and ranked higher than published TA genes. Finally, YbaQ demonstrates near perfect identity with the profile corresponding to VapB antitoxins, but has no physically close partner, so it most likely is a solitary antitoxin, the first such to be reported in *E. coli*.

Ten previously undescribed TA systems were identified in the four replicons of *A. tumefaciens *(Table 2), although only the two chromosomes were previously studied. RASTA-Bacteria confirmed the 14 systems previously reported and identified 5 additional (orphan) loci on the circular chromosome, 1 full pair and 1 orphan gene on the linear chromosome, and 2 TA systems on plasmid AT. It revealed plasmid Ti carries no plasmid addiction systems, although it does have a gene resembling *hipA *(Atu6158, GI|17939291). However, this candidate is substantially shorter than its reference, such that it is unlikely to be functional, and it is almost 60 kb away from any possible *hipB *candidate.

We also assessed the sensitivity of our tool by examining genomes containing many TA loci, including that of *N. europaea*, reported to have no less than 45 TA loci, representing 88 genes. The RASTA-Bacteria scan of the genome of *N. europaea *yielded high confidence scores for 76 of these previously identified genes (86%), a confidence score between 50% and 70% for 11 (12.5%) and an unranked score for 1. It identified 11 additional TA loci on the *N. europaea *chromosome, if the *hipBA *locus is taken into account. Three are clearly vapBC pairs, although one is made of two relatively short and possibly disrupted genes, raising doubt about whether this pair is functional. The NE2103/NE2104 pair gave an intermediate confidence score, but has characteristics consistent with it being a TA system. NE1375/NE1376 may well define a new MazEF-like system. Finally, three orphan *vapB *and two orphan *higA *genes were found: it would be interesting to determine whether they are silent relics of ancient systems or are still active and responsible for a function. Remarkably, all these newly identified loci map in the same regions as the previously discovered systems, reinforcing the observation that TA loci in *N. europaea *cluster in particular regions of the genome.

We also applied our tool to organisms where no TA loci had been found previously, including *L. lactis*, in which we predict ten possible TA loci, eight of which consist of an orphan gene containing a region encoding the same HTH_DNA-binding (for helix-turn-helix) profile.

Finally, the archaeum with the most TA loci was *S. tokodaii*, with 32 TA loci [39]. RASTA-Bacteria confirmed 52 of the 61 genes at these 32 TA loci (3 singletons): the STS188/ST1628 and ST2136/37 pairs gave low scores because of an extreme overlap or because of an alternative start codon causing a bias in the size scoring process. The results for five other genes cannot be interpreted with certainty, but observations in other organisms where orphan TA genes do not seem uncommon suggests that some loci predicted to be pairs might in fact belong to the single-gene loci category. Nevertheless, four additional TA loci were identified, two of them being standard pairs of the VapBC family. The TA loci are unevenly distributed through the chromosome: two regions of approximately 240 and 440 kb seem to be devoid of TA loci, and the loci appear to be clustered in particular regions (data not shown).

### Application to newly selected genomes

We performed a second round of analyses of newly selected, mostly recently published genomes (Table 3). At least three genomes from each phylogenetic branch and lifestyle were examined, except where only less complete genome sequences were available.

**Table 3:** RASTA-Bacteria predictions for newly sequenced genomes

Organism	*ccdAB*	*higBA*	*mazEF*	*parDE*	*phd/doc*	*relBE*	*vapBC*	*hipBA*	UnClass.	Total
*A. phagocytophilum*	0	0	0	0	0	0	0	0	0	0
*Arthrobacter aurescens*	0	0	0	0	0	0	0	0	0	0
*Azoarcus *sp. BH720	0	0	0	0	0	1	2	0	1	4
*Bartonella bacilliformis*	0	1	0	0	0	0	1	0	0	2
*Burkholderia xenovorans*	0	2	1	0	0	2	8	5	15	33
*C. P. amoebophila*	0	0	0	0	0	0	0	0	0	0
*Dehalococcoides *sp.	0	2	0	0	0	0	0	3	2	7
*Deinococcus geothermalis*	0	0	1	0	0	0	2	0	3	6
*Ehrlichia chaffeensis*	0	0	0	0	0	0	0	0	0	0
*Frankia alni ACN14a*	0	1	4	0	0	3	3	1	0	12
*Gramella forsetii KT0803*	1	3	1	5	0	1	1	0	0	12
*Granulibacter bethesdensis*	0	0	0	0	0	0	1	0	0	4
*Lawsonia intracellularis*	0	0	0	2	0	1	1	0	0	4
*Magnetococcus *sp.	1	6	0	4	1	1	4	0	0	17
*Methanococcoides burtonii*	0	2	1	0	1	0	0	0	1	5
*Methanospirillum hungatei*	0	2	3	0	0	7	15	0	2	30
*Mycobacterium bovis*	2	1	9	2	0	3	48	0	0	65
*Mycobacterium *sp. KMS	0	1	2	0	2	1	5	0	3	14
*Myxococcus xanthus*	0	4	0	0	0	0	0	1	3	10
*Nanoarchaeaum equitans*	0	0	0	0	0	0	0	0	0	0
*O. yellows phytoplasma*	0	0	0	0	0	0	0	0	0	0
*P. naphthalenivorans*	0	2	2	1	1	4	10	4	1	25
*Pyrobaculum islandicum*	0	1	0	0	0	0	2	0	2	5
*Shewanella *sp.	0	1	0	2	0	0	0	1	3	8
*Thermofilum pendens*	0	0	0	0	0	0	0	0	0	0
*Trichodesmium erythraeum*	0	0	2	2	0	0	0	1	1	6
*Wigglesworthia*	0	0	0	0	0	0	0	0	0	0
*Wolbachia*	0	0	0	0	0	7	0	0	0	7

Unclass., unclassified.

The analysis of both first and second rounds combined allowed us to confirm that there is no correlation between number of TA loci and total genome size, although the size of 1 Mb seems to represent some sort of threshold (Additional data file 1): in genomes below this size, no canonical TA systems were detected. We found no relationship between TA loci content and bacterial shape (for example rod, coccus, filament), respiratory system (aerobic or anaerobic), G+C content, presence of one or several replicons, or generation time (data not shown).

No evident association can be established between the number of TA loci and membership of a phylum (Additional data file 2) or lifestyle (Additional data file 3). The only 'free-living' organisms that do not have TA loci are *Thermofilum pendens *and *Prochlorococcus marinus*, although a possible 'pseudogenic' locus was detected in *P. marinus *strain MIT9303. The 13 other TA-free genomes are small (<1 Mb) and all correspond to obligate host-associated bacteria. In this study, the genomes of four obligate intracellular bacteria were newly discovered to carry TA systems (*Wolbachia*, *Bartonella*, *Lawsonia *and *Azoarcus*), in apparent contradiction of the hypothesis according to which endosymbiotic organisms tend to be free from TA systems. However, this hypothesis is consistent with the genome reduction theory, and quite a few studies show that chromosomal TA genes, at least, can be deleted (knockout mutants in *E. coli*) without any detectable impairment of the bacteria [15,16,52]. This assumption that TA systems have been lost during the process of genome reduction is also supported by the failure of RASTA-Bacteria to find any TA systems in the genomes of organelles, including the mitochondria (*Homo sapiens*, *Mus musculus*, *Ostreococcus tauri*) and chloroplast (*Ostreococcus tauri*, *Lotus japonicum*, *Medicago truncatula*) (data not shown).

An attempt to classify TA loci into families led to the observation that the largest family is *vapBC *(37% percent of the 532 loci found in this study), despite members of this family being absent from firmicutes and cyanobacteria. The other families each have similar numbers of representatives (*higBA*, 10% of the TA systems identified; *mazEF*, 12%; *relBE*, 12%; *hipBA*, 7%; *parDE*, 7%; and *unclassified*, 11%) except *phd/doc *and *ccdAB*, which are rarer (3% and 0.009%, respectively). Only the *parDE *family was confirmed to be confined to the bacterial domain: it was not found in archaea. Some organisms are 'monofamily', for example *Wolbachia *sp., which has seven *relBE *systems, whereas *Polaromonas naphthalenivorans *or *N. europeae*, two betaproteobacteria, contain members of all nine families (Tables 2 and 3).

The raw result tables along with selected candidates corresponding to the pre-computed genomes described in the article can be accessed from the designated section of the website [41].

## Discussion

Exhaustive high-quality annotation is an essential part of exploiting the increasing number of genome sequences available, particularly of prokaryotes. One common problem of bacterial genome annotation is the frequent omission of sORFs, especially when they present limited or no sequence homology. The more problematic consequences of this omission include the underestimation of the number of ORFs and the loss of information concerning the presence of essential functions. This is particularly the case for TA genes, which encode small proteins involved in a large variety of essential bacterial functions, especially stress physiology and programmed cell death. Indeed, these systems open new horizons for antibiotic treatments, as they constitute promising targets.

Therefore, we have developed RASTA-Bacteria, a new and unique web-based annotation tool that searches, evaluates and stores TA modules in any prokaryotic sequenced genome. The simple interface allows biologists to identify TA genes quickly and reliably, whether they be organized in pairs or isolated. Although TA classification is still problematic, functional assignment is suggested through domain identification. The power of RASTA-Bacteria in annotating putative TA modules stems from it combining detection of function, using a new knowledge-based database (TAcddb), and genomic context of the genes (small and paired).

No absolute rule can be inferred for exact prediction of the number and nature of TA loci in the genome of a bacterium based only on its characteristics, such as its phylum or ecosystem. The search for TA genes must thus be carried out *de novo *for each genome (or even each strain and each replicon), making RASTA-Bacteria a potentially valuable tool. The results described in the present study illustrate how satisfactorily the tool performs in terms of TA gene-finding accuracy (compared to earlier annotations by Pandey *et al*. [39]). In addition, the efficiency of RASTA-Bacteria is independent of genome architecture (it works with linear and circular chromosomes from 9 to less than 1 Mb, megaplasmids, plasmids), G+C content, bacterial classification (Gram+ and Gram- Eubacteria, Archaea), and lifestyle (free living, symbiont, endosymbiont). Furthermore, RASTA-Bacteria proved to be functional for eukaryotic sequences. It is thus conceivable that our tool will bring new insight to the TA world.

Our study also shows that TA systems are more various and numerous than initially reported by Pandey *et al*. [39], with a maximum of 65 loci in *Mycobacterium bovis *(Table 3). We also found a significant number of genes, encoding potential toxins or antitoxins, with no partner mapping in their vicinity. It is difficult to evaluate whether or not this phenomenon is the result of classical TA system breakage with loss of functionality of the subsequent orphan genes, or if these independent elements have evolved towards new functionalities, or even if distant interconnection is possible in the TA world. These solitary genes are not eliminated from the screening process and will be, like genes in pairs, subject to biological validations, as some ORFs that have been scored may in fact well be pseudogenes. An interesting future development of RASTA-Bacteria would be to add a 'pseudogene' field to the table.

Although the design of the RASTA-bacteria tool can sometimes lead to bias when assessing the pairing and/or length properties, it is also interesting that we found many longer proteins (between 350 and 500 amino acids) displaying similarity with TA domains throughout our study (data not shown). These findings are confusing, as they could either be the consequence of the ability of TA genes to invade other genes, and thus another argument in favor of their genetic mobility, or of a possible fusion process among toxin and antitoxin genes.

One of the most important improvements of RASTA-bacteria would be the refinement of the automatic functional classification of TA systems. Indeed, TA proteins are currently classified into rather vague classes or subclasses in the result tables, with generic domains such as the PIN domain: more precise classification of the candidates presently requires manual analysis. RASTA-Bacteria also identified numerous candidates displaying the HTH-DNA binding domains. When adjacent to an unambiguous toxin domain, these signatures could be clearly interpreted, but in some cases the identified candidates seem to constitute an orphan gene. It will thus be interesting to determine whether these loci are indeed true TA loci, or whether they constitute a new uncharacterized family of 'classical' regulators. In any case we hope that RASTA-bacteria will help biologists with bacterial TA functional characterization, which will in turn allow us to improve our algorithm by expanding our knowledge and database.

## Additional data files

The following additional data are available with the online version of this paper. Additional data file 1 is a figure showing the number of TAs in genomes as a function of size. Additional data file 2 is a figure showing the number of TAs in genomes with respect to phylogeny classification. Additional data file 3 is a figure showing the number of TAs in genomes with respect to life style.

## Supplementary Material

Additional data file 1 Number of TAs in genomes as a function of size. Click here for file

Additional data file 2 Number of TAs in genomes with respect to phylogeny classification. Click here for file

Additional data file 3 Number of TAs in genomes with respect to life style. Click here for file
