# Supplementary figures and images for: RASTA-Bacteria: a web-based tool for identifying toxin-antitoxin loci in prokaryotes
Source: Genome Biol. 2007 Aug 1;8(8):R155. doi: 10.1186/gb-2007-8-8-r155 (PMC2374986; doi:10.1186/gb-2007-8-8-r155)

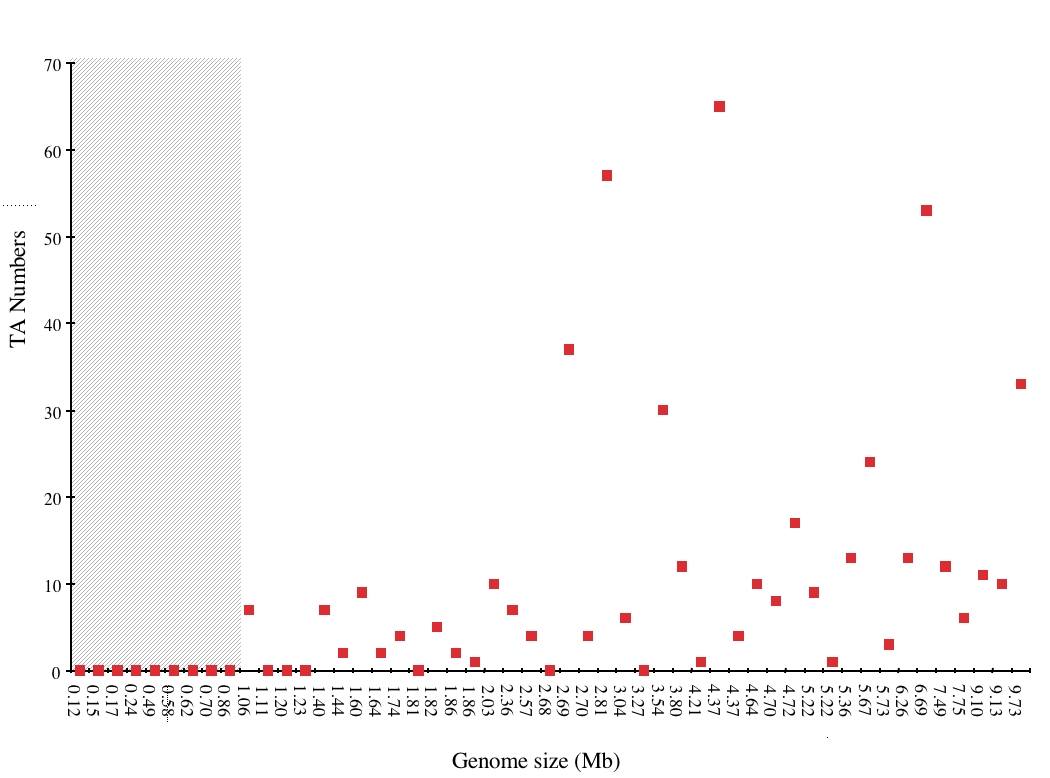

Supplement: Additional data file 1 — Number of TAs in genomes as a function of size. [file gb-2007-8-8-r155-S1.tiff]

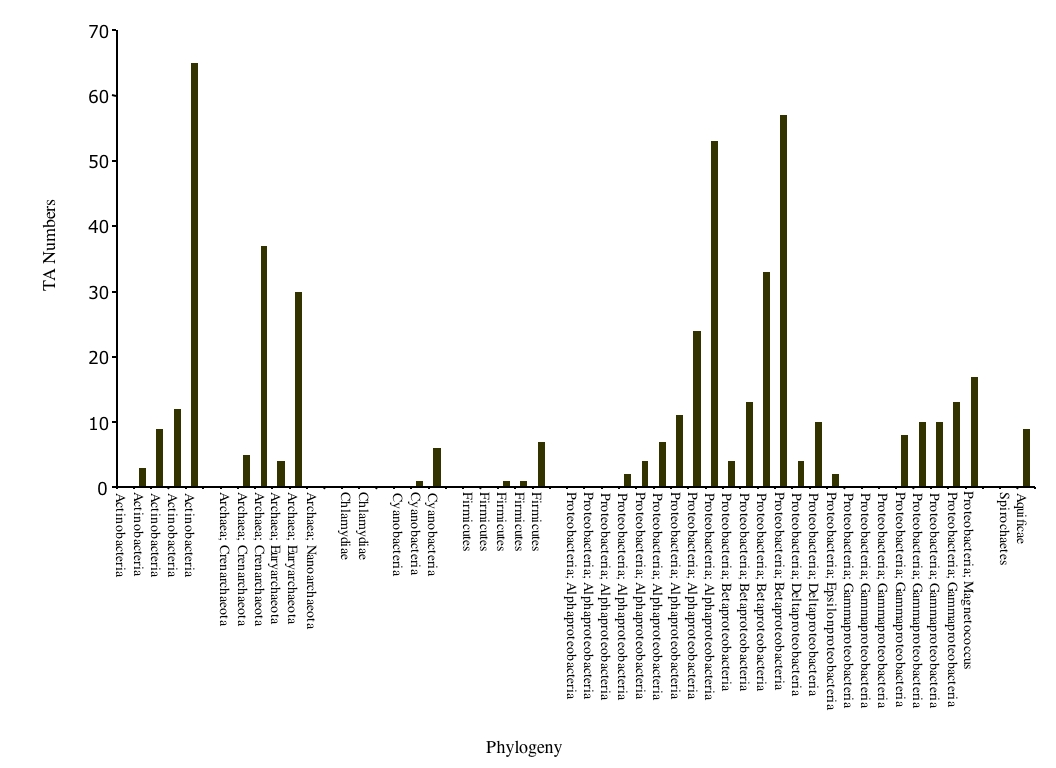

Supplement: Additional data file 2 — Number of TAs in genomes with respect to phylogeny classification. [file gb-2007-8-8-r155-S2.tiff]

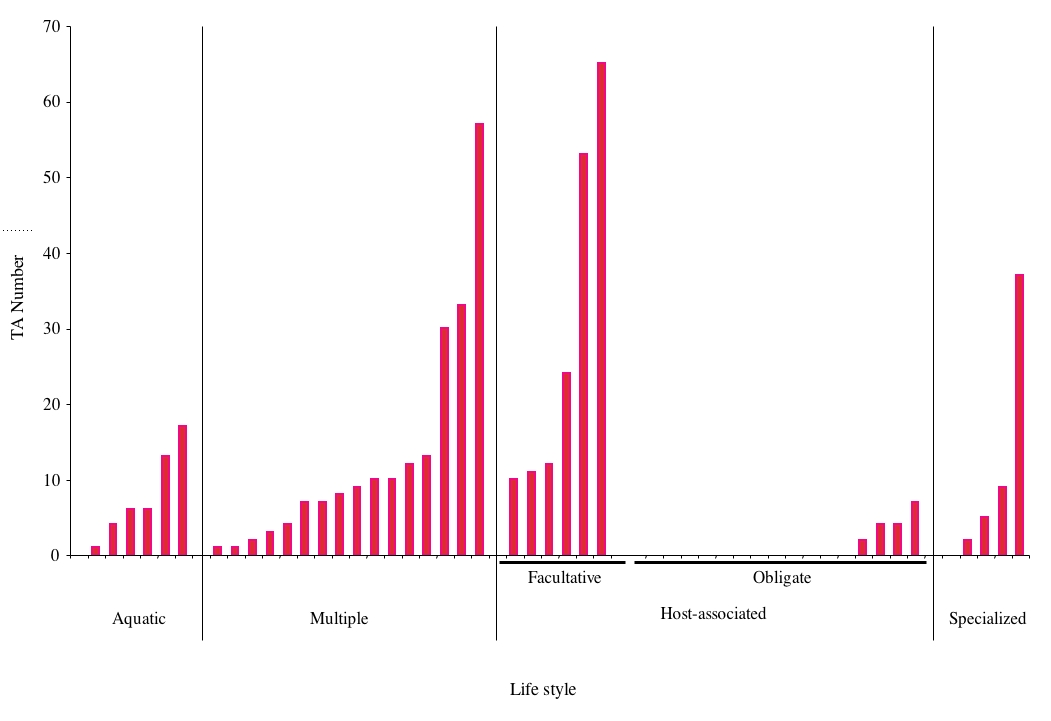

Supplement: Additional data file 3 — Number of TAs in genomes with respect to life style. [file gb-2007-8-8-r155-S3.tiff]
